# The Impact of Diabetes Mellitus on the Second Primary Malignancies in Colorectal Cancer Patients

**DOI:** 10.3389/fonc.2020.573394

**Published:** 2021-01-28

**Authors:** Jana Halamkova, Tomas Kazda, Lucie Pehalova, Roman Gonec, Sarka Kozakova, Lucia Bohovicova, Ondrej Slaby, Regina Demlova, Marek Svoboda, Igor Kiss

**Affiliations:** ^1^ Department of Comprehensive Cancer Care, Masaryk Memorial Cancer Institute, Brno, Czechia; ^2^ Department of Comprehensive Cancer Care, Faculty of Medicine, Masaryk University, Brno, Czechia; ^3^ Department of Medical Ethics, Faculty of Medicine, Masaryk University, Brno, Czechia; ^4^ Department of Radiation Oncology, Masaryk Memorial Cancer Institute, Brno, Czechia; ^5^ Department of Radiation Oncology, Faculty of Medicine, Masaryk University, Brno, Czechia; ^6^ Institute of Health Information and Statistics of the Czech Republic, Prague, Czechia; ^7^ Faculty of Medicine, Masaryk University, Brno, Czechia; ^8^ Department of Pharmacy, Masaryk Memorial Cancer Institute, Brno, Czechia; ^9^ Central European Institute of Technology, Molecular Oncology II-Solid Cancer, Masaryk University, Brno, Czechia; ^10^ Department of Pharmacology, Faculty of Medicine, Masaryk University, Brno, Czechia; ^11^ Clinical Trial Unit, Masaryk Memorial Cancer Institute, Brno, Czechia

**Keywords:** diabetes mellitus, second primary malignancies, second primary neoplasms, multiple primary neoplasms, colorectal cancer, cancer survivors

## Abstract

**Introduction:**

All colorectal cancer (CRC) survivors have an increased risk of developing second primary malignancies (SPMs). The association between diabetes mellitus (DM) and the risk of cancer is well known. However, the role of DM and its therapy in the development of SPMs in CRC patients is not well described.

**Methods:**

In this single-institutional retrospective analysis we identified 1,174 colorectal carcinoma patients, median follow-up 10.1 years, (median age 63 years, 724 men). All patients over 18 years with histologically confirmed CRC who were admitted in the period 1.1. 2003- 31.12.2013 and followed-up till 31.12. 2018 at the Masaryk Memorial Cancer Institute (MMCI) were screened for eligibility. The exclusion criteria were CRC diagnosed at autopsy, lost to follow-up and high risk of development of SPMs due to hereditary cancer syndrome. Tumours are considered multiple primary malignancies if arising in different sites and/or are of a different histology or morphology group. Comparisons of the basic characteristics between the patients with SPM and the patients without SPM were performed as well as comparison of the occurrence of SPMs by the site of diagnosis between the DM and non-DM cohorts and survival analyses.

**Results:**

A SPM was diagnosed in 234 (20%) patients, DM in 183 (15%) patients. DM was diagnosed in 22.6% of those with SPM vs. in 13.8% of those without SPM (p=0.001). The most common types of SPMs in DM patients were other CRC, kidney, lung, bladder and nonmelanoma skin cancer, but only carcinoma of the liver and bile duct tracts was significantly more common than in the group without DM. Although breast cancer was the second most common in the group with DM, its incidence was lower than in the group without DM, as well as prostate cancer. A significantly higher incidence of SPMs was found in older CRC patients (≥ 65 years) and in those with lower stage colon cancer and DM. No significant difference in DM treatment between those with and without a SPM was observed including analysis of type of insulin.

**Conclusion:**

CRC patients with diabetes mellitus, especially those with older age, and early stages of colon cancer, should be screened for second primary malignancies more often than the standard population. Patients without DM have longer survival. According to the occurrence of the most common second malignancies, a clinical examination, blood count, and ultrasound of the abdomen is appropriate, together with standard breast and colorectal cancer screening, and lung cancer screening under certain conditions, and should be recommended in CRC survivors especially in patients with intercurrent DM, however the necessary frequency of screening remains unclear.

## Introduction

Colorectal carcinoma (CRC) is one of the most common malignant tumors in all western countries. Due to the success of personalized therapy and screening, mortality from this disease has been reduced in recent times. In 2015, its prevalence in the Czech Republic (third rank in incidence within Europe) reached 64 126 persons and increased by almost 40% in comparison with 2005 (1). However, the increasing number of people being cured carries the risk of development of another type of cancer. In Western countries, 17% of all cancer patients experience second primary malignancy (SPM) during their lifetime (2). CRC patients after curative resection are thought to have an additional tumor risk of up to 40% (3). For this reason, it is necessary to focus attention on the early diagnosis of other malignancies in patients with complete remission and adapt the type and timing of screening for SPMs. Primary malignancies are associated with lifestyle, environmental risk factors, and hereditary factors, in secondary tumours, treatment of previous cancer is additionally added.

The associations between diabetes mellitus (DM) and the risk of cancer is well known (4), nevertheless, the factors responsible for this relationship remain unclear. Insulin is a growth factor and major regulator of cell metabolism. Stimulation of growth is facilitated by the insulin receptor which is expressed on cancer cells in an A isoform, known by its predominant mitogenic effect which can stimulate neoplastic proliferation (5). Other factors responsible for cancer development are hyperglycemia accompanying insulin resistance leading to hyperinsulinemia, insulin-like growth factor 1, oxidative stress, and inflammation (6). Obesity which is linked to diabetes mellitus type II is responsible for an increased risk of cancer as well (7). It is hypothetized, that the type of DM treatment also plays an important role in the development of cancer (8, 9). Peroral antidiabetics (PADs) and insulin are long-term standards of care for patients with diabetes mellitus. Previously used animal insulin is currently replaced by recombinant human insulins produced by recombinant DNA technology, which use Escherichia Coli or Saccharomyces cerevisiae. In recent years, insulin glargine has acquired much attention in cancer patients. Insulin glargine (GlyA21, ArgB31, ArgB32 human insulin) is insulin produced by recombinant DNA technology using E. coli, substituting asparagine at position 21 in the A chain with glycine and adding two arginine residues to the B chain at positions 31 and 32 (10). In a large German study, a higher cancer incidence was associated with administration of glargine compared to human insulin. On the other hand, the opposite was described in other retrospective trials and a metaanalysis (11–16). It seems that observational studies describing insulin glargine as a risk factor for developing cancer have important methodological bias (17) and, thus, the importance of insulin glargine in the development of cancer remains unclear (18, 19). There is no robust evidence describing the influence of the type of production of insulin on the development of SPMs or risk of cancer.

In addition to insulin, oral antidiabetic drugs (PAD) are also used to treat diabetes with metformin being one of the most commonly prescribed. Metformin is an antihyperglycemic drug with a hypoglycemic effect without hyperglycemia, it improves insulin resistance (20) and decreases circulating insulin levels through activation of the adenosine monophosphate-activated protein kinase (AMPK) pathway (21). Antiproliferative potential is demonstrated by reduced prevalence and number of metachronous adenomas or polyps after polypectomy (22) and, thus, it is considered as a protective factor in colorectal adenomas and subsequent carcinomas (23). In multiple studies, metformin has also been identified as a drug with anticancer activity, especially in CRC (24–30).

A metaanalysis of 24 metformin studies demonstrates that metformin usage decreases cancer risk in diabetes mellitus type II patients (8) and that metformin could have a protective effect (29, 31). However, no large studies evaluating the risk of development of SPMs and type of treatment of diabetes mellitus in CRC patients are currently available.

The aim of this single-institutional retrospective analysis is the identification of SPMs in colorectal cancer patients and description of the potential relationship between the occurrence of DM, its treatment and the development of SPMs.

## Material and Methods

### Patients Selection

After approval by the institutional ethics committee (2019/1827/MOU), all patients over 18 years with histologically confirmed CRC who were admitted in the period 1.1. 2003- 31.12.2013 and followed-up till 31.12. 2018 at the Masaryk Memorial Cancer Institute (MMCI) in Brno, Czech Republic, were screened for eligibility after signing their informed consent enabling use of their personal data in the research. All patients who did not meet the exclusion criteria were included. The exclusion criteria were as follows: CRC diagnosed at autopsy, lost to follow-up and high risk of development of SPMs due to hereditary cancer syndrome (e.g., BRCA1,2, Lynch syndrome, or familial adenomatous polyposis). Basic diagnostic and treatment data including the laterality of CRC were retrieved from electronic medical records. Additional data about the type of DM, type of treatment of DM, and type of PAD or insulin therapy were obtained in patients with a diagnosis of DM. The diagnosis of DM had to precede the first malignancy.

### Second Primary Malignancies

For epidemiological studies, tumors are considered multiple primary malignancies if arising in different sites and/or are of a different histology or morphology group (32). In our study, criteria according to the SEER definition of multiple primary tumors were used: 1) tumors with ICD-O-3 histology codes that are different at the first, second or third number are multiple primaries; 2) tumors with ICD-O-3 topography codes that are different at the second and/or third characters are multiple primaries (33).

Synchronicity and multiplicity were qualified according to the rules of the International Agency for Research on Cancer (IARC) which suggest synchronous tumours to be diagnosed in an interval of less than 6 months (or metachronous if more than 6 months) and if arising in different sites (34).

### Statistical Analysis

Comparisons of the basic characteristics between the patients with SPM and the patients without SPM were summarized with counts and frequencies and tested with the Fisher exact test in case of categorical characteristics. For countinuous characteristics median, 5%–95% percentile and the Mann-Whitney test was used. The Fisher exact test was also used to test the relationship between the occurrence of SPMs on one side and the presence of DM, DM therapy, and the laterality of colorectal cancer on the other side.

Comparison of the occurrence of SPMs by the site of diagnosis between the DM and non-DM cohorts was performed by the N-1 chi-squared test. SPMs with an unknown date of diagnosis were not included in the analysis (7 cases). The national cancer registry of the Czech Republic (35) was used to compare the frequencies of sites of diagnosis in our study with the frequencies in the entire Czech population.

Kaplan-Meier curves were utilized to display the survival of the patients with colorectal cancer stratified by the occurrence of SPM and DM. 15-year survival was used as the primary endpoint. Observations with 15 or more years of follow-up were censored at 15 years. The Breslow test was used to compare the differences in survival between defined groups of patients with respect to the presence of DM and the occurrence of SPM.

## Results

### Second Primary Malignancies

In total, 1174 patients were identified and enrolled in this study. The median follow-up was 10.1 years, median age 63 years and 724 of the patients were men (62%). The other basic characteristics are summarized in **Table 1** in respect to occurance of SPM, which was diagnosed in 234 (20%) patients (**Table 2**). One secondary neoplasm was found overall in 190 (16.2%) patients, 36 (3.1%) patients suffered from two SPMs and 8 (0.7%) were treated with three SPMs (**Table 2**). A significantly higher incidence of SPMs was observed in older CRC patients and also in patients with a lower stage of CRC reflecting their better overall survival.

**Table 1:** Characteristics of colorectal cancer patients (C18–C20) stratified by the occurrence of second primary malignancies.

	No SPM (N = 940)	With SPM (N = 234)	p-value
Gender			
Men	590 (62.8%)	134 (57.3%)	0.133^1^
Women	350 (37.2%)	100 (42.7%)	
Age at CRC diagnosis			
0–44	79 (8.4%)	14 (6.0%)	0.001^1^
45–54	153 (16.3%)	21 (9.0%)	
55–64	296 (31.5%)	58 (24.8%)	
65–74	278 (29.6%)	93 (39.7%)	
75+	134 (14.3%)	48 (20.5%)	
Median (5%–95% percentile)	63 (55–70)	67 (60–73)	< 0.001^2^
Clinical stage			
Complete records	906 (96.4%)	221 (94.4%)	0.012^1^
Stage I + in situ	249 (27.5%)	68 (30.8%)	
Stage II	218 (24.1%)	67 (30.3%)	
Stage III	260 (28.7%)	61 (27.6%)	
Stage IV	179 (19.8%)	25 (11.3%)	
Occurrence of DM			
No	810 (86.2%)	181 (77.4%)	0.001
Yes	130 (13.8%)	53 (22.6%)	

^1^Fischer exact test; ^2^Mann-Whitney test; SPM, second primary malignancy; CRC, colorectal cancer.

**Table 2:** Second primary malignancies in patients with colorectal cancer (C18–C20).

Patients with CRC	Men (N = 724)	Women (N = 450)	Total (N = 1 174)
No SPM	590 (81.5%)^1^	350 (77.8%)^1^	940 (80.1%)
With SPM	134 (18.5%)^1^	100 (22.2%)^1^	234 (19.9%)
Two primary neoplasms	112 (15.5%)	78 (17.3%)	190 (16.2%)
Three primary neoplasms	18 (2.5%)	18 (4.0%)	36 (3.1%)
Four primary neoplasms	4 (0.6%)	4 (0.9%)	8 (0.7%)

^1^p-value of Fisher exact test: 0,133. SPM, second primary malignancy; CRC, colorectal cancer.

### Diabetes Mellitus

Diabetes mellitus was diagnosed in 183 (15.5%) patients. DM was diagnosed in 22.6% of those with SPM vs. in 13.8% of those without SPM (p=0.001). The type of DM treatment is summarized in **Table 3**. Oral antidiabetic drugs (PADs) alone or in combination with insulin were taken by 127 patients. No significant difference in DM treatment between those with and without SPM was observed including analysis of type of insulin and its production.

**Table 3:** Relationship between treatment of diabetes mellitus and risk of second primary malignancy in patients with colorectal cancer (C18–C20).

Therapy of DM	No SPM (N = 130)	With SPM (N = 53)	p-value
Diet	32 (24.6%)	12 (22.6%)	0.737
PAD	65 (50.0%)	30 (56.6%)	
PAD/Insulin	25 (19.2%)	7 (13.2%)	
Insulin	8 (6.2%)	4 (7.5%)	
**Therapy of DM - PAD**	**No SPM (N = 90)**	**With SPM (N = 37)**	**p-value**
Metformin	74 (82.2%)	33 (89.2%)	0.427
Other PAD	16 (17.8%)	4 (10.8%)	
**Therapy of DM - insulin**	**No SPM (N = 33)**	**With SPM (N = 11)**	**p-value**
Glargine	6 (18.2%)	2 (18.2%)	1.000
Other insulin	27 (81.8%)	9 (81.8%)	
Insulin made by recombinant DNA technology in Escherichia coli	8 (24.2%)	2 (18.2%)	1.000
Insulin made by recombinant DNA technology in Saccharomyces cerevisiae	25 (75.8%)	9 (81.8%)	

SPM, second primary malignancy; DM, diabetes mellitus; PAD, oral antidiabetics.

**Figure 1:**
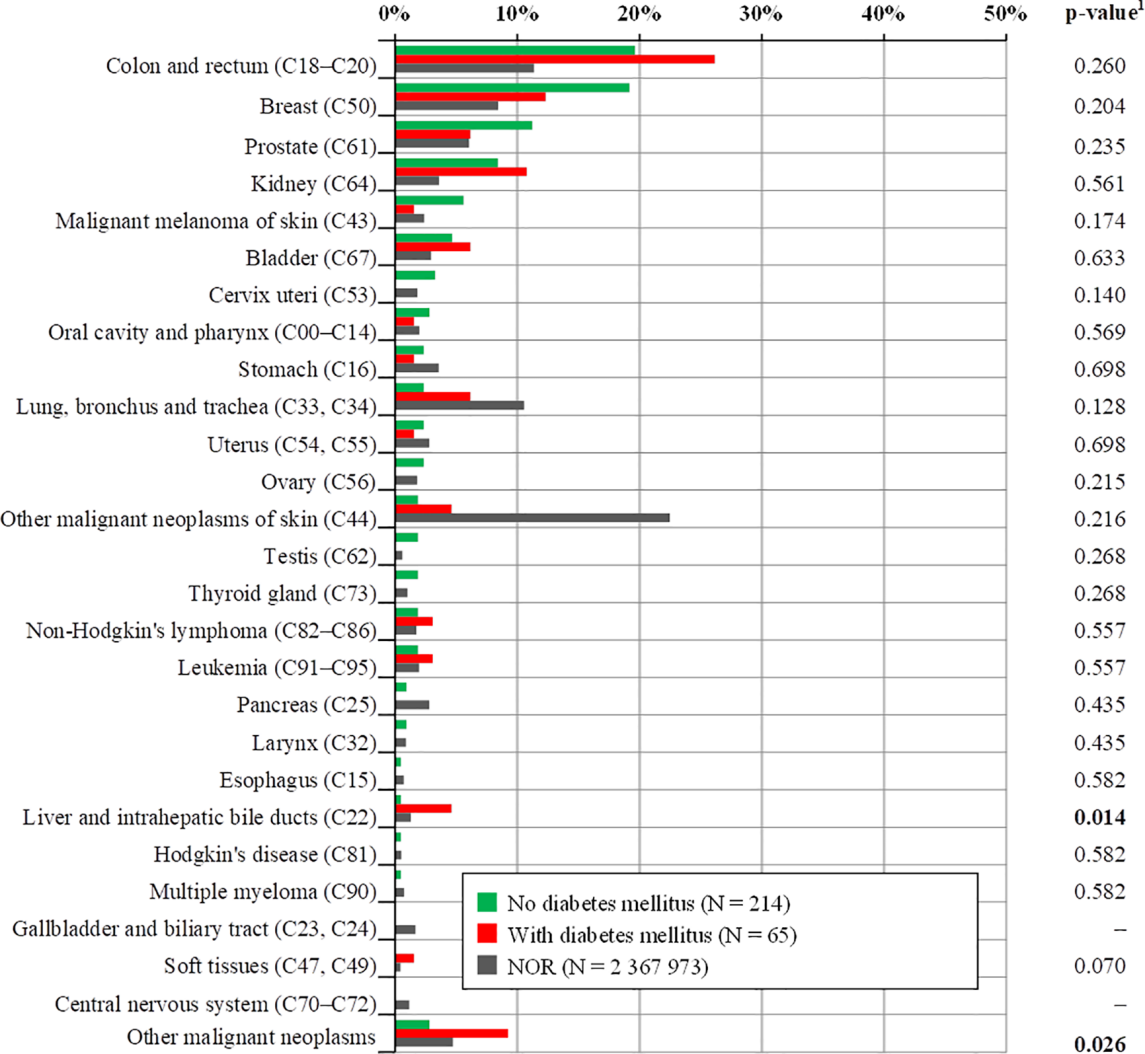
Comparison of the occurrence of total second primary malignancies with respect to diabetes mellitus. Only SPMs with known date of diagnosis were considered (date of diagnosis was not available for seven SPMs). ^1^p-value of N-1 Chi-squared test for group no diabetes mellitus and group with diabetes mellitus. SPMs, second primary malignancies; CRC, colorectal cancer, NOR, national cancer registry (1977–2017).

CRC patients with diabetes mellitus had a higher incidence of SPMs than those without DM, especially another CRC, liver and intrahepatic bile ducts, lung, nonmelanoma tumors of the skin, kidney, bladder, non-Hodgkin disease, and leukemia (**Table 4**), but except for liver and intrahepatic bile duct cancer (4.6% with DM vs. 0.5% without DM, p=0.014), a higher incidence of other SPMs was not statistically significant (**Figure 1**). Although breast cancer is the second most common in the group with DM, its incidence is lower than in the group without DM, as well as prostate cancer. Statistical significance of a group of other malignant neoplasms is biased by multiple diagnostic units and is listed in **Table 5**.

**Table 4:** Second primary malignancies by the site of diagnosis stratified by the occurrence of diabetes mellitus.

	No diabetes mellitus (N = 214)	With diabetes mellitus (N = 65)	All malignant neoplasms according to NOR (N = 2,367,973)
Oral cavity and pharynx (C00–C14)	6 (2.8%)	1 (1.5%)	47,097 (2.0%)
Esophagus (C15)	1 (0.5%)	0 (0.0%)	16,943 (0.7%)
Stomach (C16)	5 (2.3%)	1 (1.5%)	84,738 (3.6%)
Colon and rectum (C18–C20)	42 (19.6%)	17 (26.2%)	268,753 (11.3%)
Liver and intrahepatic bile ducts (C22)	1 (0.5%)	3 (4.6%)	30,775 (1.3%)
Gallbladder and biliary tract (C23, C24)	0 (0.0%)	0 (0.0%)	39,697 (1.7%)
Pancreas (C25)	2 (0.9%)	0 (0.0%)	65,789 (2.8%)
Larynx (C32)	2 (0.9%)	0 (0.0%)	21,055 (0.9%)
Lung, bronchus and trachea (C33, C34)	5 (2.3%)	4 (6.2%)	249,926 (10.6%)
Malignant melanoma of skin (C43)	12 (5.6%)	1 (1.5%)	56,372 (2.4%)
Other malignant neoplasms of skin (C44)	4 (1.9%)	3 (4.6%)	532,199 (22.5%)
Soft tissues (C47, C49)	0 (0.0%)	1 (1.5%)	10,358 (0.4%)
Breast (C50)	41 (19.2%)	8 (12.3%)	199,562 (8.4%)
Cervix uteri (C53)	7 (3.3%)	0 (0.0%)	43,373 (1.8%)
Uterus (C54, C55)	5 (2.3%)	1 (1.5%)	66,192 (2.8%)
Ovary (C56)	5 (2.3%)	0 (0.0%)	42,593 (1.8%)
Prostate (C61)	24 (11.2%)	4 (6.2%)	142,994 (6.0%)
Testis (C62)	4 (1.9%)	0 (0.0%)	14,440 (0.6%)
Kidney (C64)	18 (8.4%)	7 (10.8%)	85,270 (3.6%)
Bladder (C67)	10 (4.7%)	4 (6.2%)	69,826 (2.9%)
Central nervous system (C70–C72)	0 (0.0%)	0 (0.0%)	27,516 (1.2%)
Thyroid gland (C73)	4 (1.9%)	0 (0.0%)	23,545 (1.0%)
Hodgkin’s disease (C81)	1 (0.5%)	0 (0.0%)	12,082 (0.5%)
Non-Hodgkin’s lymphoma (C82–C86)	4 (1.9%)	2 (3.1%)	41,122 (1.7%)
Multiple myeloma (C90)	1 (0.5%)	0 (0.0%)	17,252 (0.7%)
Leukemia (C91–C95)	4 (1.9%)	2 (3.1%)	46,717 (2.0%)
Other malignant neoplasms	6 (2.8%)	6 (9.2%)	111,787 (4.7%)

Only SPMs with known date of diagnosis were considered (date of diagnosis was not available for seven SPMs).

SPM, second primary malignancy; CRC, colorectal cancer, NOR, national cancer registry (1977–2017).

**Table 5:** Other malignant neoplasms as second primary malignancies in detail.

	No diabetes mellitus (N = 6)	With diabetes mellitus (N = 6)
Small intestine (C17)	2 (33.3%)	3 (50.0%)
Anus and anal canal (C21)	1 (16.7%)	1 (16.7%)
Thymus (C37)	0 (0.0%)	1 (16.7%)
Penis (C60)	1 (16.7%)	1 (16.7%)
Eye and adnexa (C69)	1 (16.7%)	0 (0.0%)
Malignant immunoproliferative diseases (C88)	1 (16.7%)	0 (0.0%)

According to the date of diagnosis of SPMs, patients were divided into three groups (before the development of CRC, synchronous and metachronous SPM). These and individual SPMs according to DM are summarized in **Table 6**. In patients with DM, there was a statistically significant difference in laterality of initial CRC cancer between the SPM and non-SPM groups (**Table 7**). In the SPM group, a higher proportion of cancer of the righ colon and left colon and, conversely, a lower proportion of rectal cancer compared to the group without SPM was observed (p= 0.014). Patients with rectal cancer and DM had the smallest probability of developing SPM. The transverse colon (C18.4) was excluded from the laterality assessment, due to the difficult assignment into the group for the right or left colon, only by ICD-O-3 topography codes.

**Table 6:** Second primary malignancies by the site of diagnosis stratified by the occurrence of diabetes mellitus.

	No diabetes mellitus (N = 214)				With diabetes mellitus (N = 65)				All malignant neoplasms according to NOR (N = 2,367,973)
	SPM before^1^ the first CRC(N = 82)	SPM synchronously^2^ with the first CRC (N = 59)	SPM after^3^ the first CRC(N = 73)	Total SPM(N = 214)	SPM before^1^ the first CRC(N = 22)	SPM synchronously^2^ with the first CRC (N = 27)	SPM after^3^ the first CRC(N = 16)	Total SPM(N = 65)	
Oral cavity and pharynx (C00–C14)	3 (3,7%)	1 (1,7%)	2 (2,7%)	6 (2,8%)	0 (0,0%)	0 (0,0%)	1 (6,3%)	1 (1,5%)	47,097 (2.0%)
Esophagus (C15)	0 (0,0%)	0 (0,0%)	1 (1,4%)	1 (0,5%)	0 (0,0%)	0 (0,0%)	0 (0,0%)	0 (0,0%)	16,943 (0.7%)
Stomach (C16)	1 (1,2%)	1 (1,7%)	3 (4,1%)	5 (2,3%)	0 (0,0%)	1 (3,7%)	0 (0,0%)	1 (1,5%)	84,738 (3.6%)
Colon and rectum (C18–C20)	0 (0,0%)	28 (47,5%)	14 (19,2%)	42 (19,6%)	0 (0,0%)	13 (48,1%)	4 (25,0%)	17 (26,2%)	268,753 (11.3%)
Liver and intrahepatic bile ducts (C22)	0 (0,0%)	1 (1,7%)	0 (0,0%)	1 (0,5%)	0 (0,0%)	1 (3,7%)	2 (12,5%)	3 (4,6%)	30,775 (1.3%)
Gallbladder and biliary tract (C23, C24)	0 (0,0%)	0 (0,0%)	0 (0,0%)	0 (0,0%)	0 (0,0%)	0 (0,0%)	0 (0,0%)	0 (0,0%)	39,697 (1.7%)
Pancreas (C25)	1 (1,2%)	1 (1,7%)	0 (0,0%)	2 (0,9%)	0 (0,0%)	0 (0,0%)	0 (0,0%)	0 (0,0%)	65,789 (2.8%)
Larynx (C32)	2 (2,4%)	0 (0,0%)	0 (0,0%)	2 (0,9%)	0 (0,0%)	0 (0,0%)	0 (0,0%)	0 (0,0%)	21,055 (0.9%)
Lung, bronchus and trachea (C33, C34)	0 (0,0%)	0 (0,0%)	5 (6,8%)	5 (2,3%)	2 (9,1%)	2 (7,4%)	0 (0,0%)	4 (6,2%)	249,926 (10.6%)
Malignant melanoma of skin (C43)	5 (6,1%)	3 (5,1%)	4 (5,5%)	12 (5,6%)	1 (4,5%)	0 (0,0%)	0 (0,0%)	1 (1,5%)	56,372 (2.4%)
Other malignant neoplasms of skin (C44)	2 (2,4%)	1 (1,7%)	1 (1,4%)	4 (1,9%)	0 (0,0%)	2 (7,4%)	1 (6,3%)	3 (4,6%)	532,199 (22.5%)
Soft tissues (C47, C49)	0 (0,0%)	0 (0,0%)	0 (0,0%)	0 (0,0%)	1 (4,5%)	0 (0,0%)	0 (0,0%)	1 (1,5%)	10,358 (0.4%)
Breast (C50)	26 (31,7%)	6 (10,2%)	9 (12,3%)	41 (19,2%)	8 (36,4%)	0 (0,0%)	0 (0,0%)	8 (12,3%)	199,562 (8.4%)
Cervix uteri (C53)	6 (7,3%)	1 (1,7%)	0 (0,0%)	7 (3,3%)	0 (0,0%)	0 (0,0%)	0 (0,0%)	0 (0,0%)	43,373 (1.8%)
Uterus (C54, C55)	4 (4,9%)	0 (0,0%)	1 (1,4%)	5 (2,3%)	1 (4,5%)	0 (0,0%)	0 (0,0%)	1 (1,5%)	66,192 (2.8%)
Ovary (C56)	1 (1,2%)	0 (0,0%)	4 (5,5%)	5 (2,3%)	0 (0,0%)	0 (0,0%)	0 (0,0%)	0 (0,0%)	42,593 (1.8%)
Prostate (C61)	12 (14,6%)	6 (10,2%)	6 (8,2%)	24 (11,2%)	1 (4,5%)	1 (3,7%)	2 (12,5%)	4 (6,2%)	142,994 (6.0%)
Testis (C62)	4 (4,9%)	0 (0,0%)	0 (0,0%)	4 (1,9%)	0 (0,0%)	0 (0,0%)	0 (0,0%)	0 (0,0%)	14,440 (0.6%)
Kidney (C64)	1 (1,2%)	8 (13,6%)	9 (12,3%)	18 (8,4%)	1 (4,5%)	3 (11,1%)	3 (18,8%)	7 (10,8%)	85,270 (3.6%)
Bladder (C67)	2 (2,4%)	1 (1,7%)	7 (9,6%)	10 (4,7%)	1 (4,5%)	2 (7,4%)	1 (6,3%)	4 (6,2%)	69,826 (2.9%)
Central nervous system (C70–C72)	0 (0,0%)	0 (0,0%)	0 (0,0%)	0 (0,0%)	0 (0,0%)	0 (0,0%)	0 (0,0%)	0 (0,0%)	27,516 (1.2%)
Thyroid gland (C73)	1 (1,2%)	0 (0,0%)	3 (4,1%)	4 (1,9%)	0 (0,0%)	0 (0,0%)	0 (0,0%)	0 (0,0%)	23,545 (1.0%)
Hodgkin’s disease (C81)	1 (1,2%)	0 (0,0%)	0 (0,0%)	1 (0,5%)	0 (0,0%)	0 (0,0%)	0 (0,0%)	0 (0,0%)	12,082 (0.5%)
Non-Hodgkin’s lymphoma (C82–C86)	3 (3,7%)	1 (1,7%)	0 (0,0%)	4 (1,9%)	2 (9,1%)	0 (0,0%)	0 (0,0%)	2 (3,1%)	41,122 (1.7%)
Multiple myeloma (C90)	1 (1,2%)	0 (0,0%)	0 (0,0%)	1 (0,5%)	0 (0,0%)	0 (0,0%)	0 (0,0%)	0 (0,0%)	17,252 (0.7%)
Leukemia (C91–C95)	2 (2,4%)	0 (0,0%)	2 (2,7%)	4 (1,9%)	1 (4,5%)	0 (0,0%)	1 (6,3%)	2 (3,1%)	46,717 (2.0%)
Other malignant neoplasms	4 (4,9%)	0 (0,0%)	2 (2,7%)	6 (2,8%)	3 (13,6%)	2 (7,4%)	1 (6,3%)	6 (9,2%)	111,787 (4.7%)

Only SPMs with known date of diagnosis were considered (date of diagnosis was not available for seven SPMs).

^1^diagnosed 6 or more months before the first CRC in the patient.

^2^diagnosed within 6 months before or after the first CRC in the patient.

^3^diagnosed 6 or more months after the first CRC in the patient.

SPM, second primary malignancy; CRC, colorectal cancer, NOR, national cancer registry (1977–2017).

**Table 7:** Relationship between second primary malignancies and laterality of colorectal cancer stratified by the occurrence of diabetes mellitus excluding patients with C18.4 (transverse colon).

Laterality	No diabetes mellitus (N = 955)			With diabetes mellitus (N = 174)		
	No SPM(N = 810)	With SPM(N = 181)	p-value of Fisher exact test	No SPM (N = 130)	With SPM (N = 53)	p-value of Fisher exact test
Right colon (C18.0–C18.3)	137 (17.5%)	35 (20.2%)	0.651	19 (15.3%)	15 (30.0%)	**0.014**
Left colon (C18.5–C19)	216 (27.6%)	48 (27.7%)		34 (27.4%)	18 (36.0%)	
Rectum (C20)	429 (54.9%)	90 (52.0%)		71 (57.3%)	17 (34.0%)	

SPM, second primary malignancy.

Bold values emphasize statistical significance.

**Figure 2:**
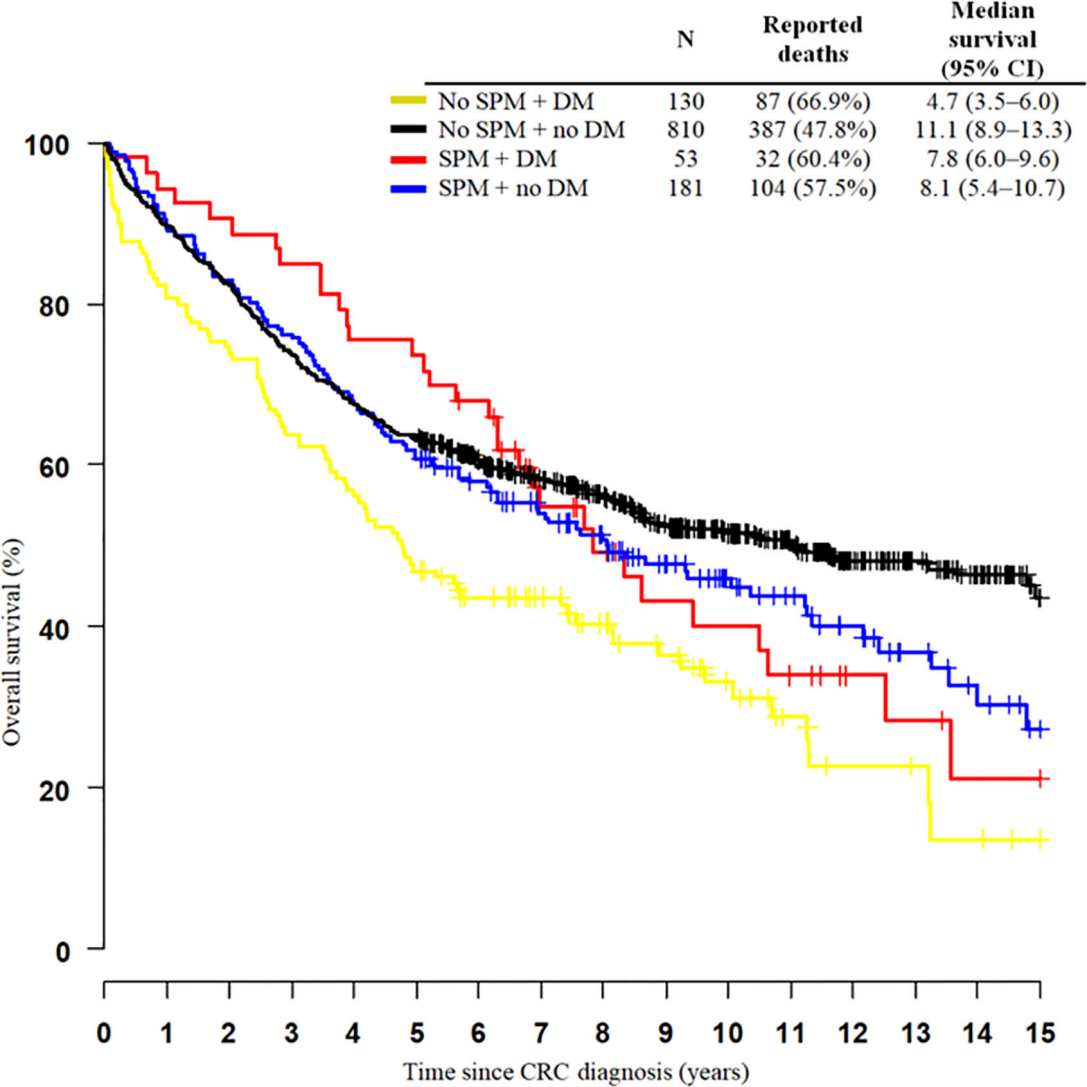
Kaplan-Meier curves of 15-year survival among colorectal cancer patients (C18–C20) stratified by the occurrence of multiple primary neoplasms and diabetes mellitus. SPM, second primary neoplasm; DM, diabetes mellitus; CI, confidence interval.

Overall survival (OS) differed according to the occurence of SPM and DM (Breslow test p=0.001). Patients without SPMs and with DM have shorter OS (median 4.7 years) than patients with SPMs and DM (median 7.8 years). Patients without SPMs have shorter survival, probably because of the poor prognosis of primary CRC in combination with DM than those with SPMs and DM, who have early stages of CRC, longer survival, and a higher probability of development SPMs. Patients without DM have longer survival, than those with DM. DM seems to be an important factor for survival. Patients without DM have a similar survival estimation for both groups (with or without SPMs) during the first 5 years, after which patients without diabetes and SPMs live longer (**Figure 2** and **Table 8**).

**Table 8:** P-values of Breslow test for colorectal cancer patients (C18–C20) stratified by the occurrence of second primary malignancy and diabetes mellitus.

	No SPM + DM	No SPM + no DM	SPM + DM	SPM + no DM	Overall comparison
**No SPM + DM**	–	**<0.001**	**0.009**	**0.007**	**0.001**
**No SPM + no DM**	**<0.001**	–	0.721	0.468	
**SPM + DM**	**0.009**	0.721	–	0.438	
**SPM + no DM**	**0.007**	0.468	0.438	–	

SPM, second primary malignancy; DM, diabetes mellitus.

Bold values emphasize statistical significance.

## Discussion

In patients with CRC and a history of DM, a higher incidence of second primary malignancies compared with CRC patients without DM was observed in this large retrospective study with more than a 10 year follow up. Identifying the group of patients with CRC at higher risk of developing a SPM, and analyzing their type and timing is essential for clinical practice and development of long-term management, especially with increasing prevalence associated with better treatment and screening programes. This group of patients with SPMs is usually excluded from clinical trials, and available information about their OS or other related factors are limited. Recently, an online competing-risk nomogram was released (http://biostat.fudan.edu.cn/crc) (36), however, without DM listed as a risk factor.

Considering the general biology of carcinogenesis, each primary malignancy is associated with the occurrence of secondary malignancies, but the type of SPMs does not have to be the same. For example, breast cancer survivors often developed secondary breast cancer and colorectal cancer (37) and lung cancer is associated with the occurrence of other tumors of the lung, head and neck and the genitourinary tract (38). According to Jia et al., CRC survivors with an older age, male sex, with localized disease, and treatment with surgery are at high risk of developing SPMs (36, 39). A high incidence of the SPM in older patients is probably due to the long exposition of toxic substances in the environment during the longer life of these people. Also in patients with DM there was a higher incidence of SPMs, and DM was an independent risk factor for the occurrence of SPMs in gastric cancer patients (40). The higher incidence of liver and intrahepatic bile duct cancer in our analysis is in contrast with Broman et al´s. study, where the incidence of these tumors was lower than expected, it is probably due to our detailed information from source documentation, where hepatic lesions are well diagnosed which is not the case in Broman´s analysis, where possible misclassification of primary liver tumors as colorectal metastases in patients with a history of CRC were admitted (41). Relationship between diabetes and risk of second primary contralateral breast cancer was described in the study Li et al. Women with DM had a 2.2-fold increased risk of contralateral breast cancer than non-diabetics patients (42). Diabetes mellitus was identified as a potential risk factor for development of SPMs in cholangiocarcinoma patients (43).

The risk of development of a SPM is inherently associated with survival after treatment of a primary malignancy which is limited in a more advanced local tumor or even in primarily metastatic disease. In concordance with our results, an analysis by Jia et al. (36) shows that patients with SPMs have better OS in the first 10 years and thereafter, they had worse survival than patients without SPMs. In our study OS was better in the first 7 years for patients with SPMs and DM but, thereafter was worse than SPMs without DM, patients without SPMs and with DM had the worst OS.

In addition to DM itself, its treatment, antidiabetic therapy, was described as a risk factor for developing cancer and it seems that antidiabetic treatment may also play a role in carcinogenesis. In previously published literature, insulin use has been associated with increased and metformin with decreased incidence of colorectal cancer (44). Among insulin users, an increased risk of breast cancer was reported (45). Patients treated with metformin have no lower probability of SPMs incidence in our group of patients. In concordance with our results, in head and neck cancer metformin does not show a protective effect on the development of SMPs (46), on the other hand, in the development of primary pancreatic cancer this relationship was significant (47). Although long-term use of metformin appears to have the effect of reducing the incidence of CRC and its progression (48), it appears to have no effect on the incidence of secondary malignancies in CRC patients, as we have shown. In one previous study users of insulin glargine and users of other insulin analogs had a lower risk of cancer in general than those using human insulin (49), but on the other hand, an increased risk of breast cancer in users of insulin glargine in comparison with users of human insulin was found (50). For users of glargine insulin compared to users of non-glargine insulin, a decreased risk of colon cancer, as well as a marginally significant increased risk of breast cancer and prostate cancer, was observed (9, 51). However, in some studies, the effect on cancer development has not been confirmed (14). It has even been previously described that serum of patients treated by insulin glargine is more mitogenic to a breast cancer cell than those treated by other types of insulins (52). Recently, a higher risk of development of cancer was not found in a patient treated by insulin glargine or detemir compared with human insulin (53) and according to our results, insulin glargine was not associated with a higher risk of SPMs in our group of patients.

An inherent limitation of this study is related to its retrospective nature, which is similar to all other studies dealing with this issue. The same reason limits availability of some other data which may be related to the risk of SPM, such as obesity, which increases the risk of malignancy (54) as well as information on alcohol use, smoking, diet, sports activity, and lifestyle (55, 56). Although one may assume that patients with DM have mostly uniform diet, this and other information was not available for the majority of our patient´s cohort and has a significant impact on cancer development. Due to the length of follow-up and changes in the treatment strategy for both CRC and diabetes mellitus, patients with a more recent diagnosis of CRC could survive longer, and their SPMs may not have been detected yet, despite the long follow-up of our group of patients. The strengths of our study include use of a well-characterized and population-based cohort of CRC survivors, patient characteristics, and treatment with extensive follow-up, detailed information on the incidence of SPMs in CRC patients from the source documentation, review of medical charts, and detailed information about antidiabetic medication of patients.

The better identification of risk groups of patients is important for clinicians, health care providers, and health insurance companies. From our analysis it has arisen that CRC patients stage I or II with diabetes mellitus have a higher incidence of SPMs, especially second colon and rectal cancer, liver and intrahepatic bile ducts, lung, nonmelanoma tumors of the skin, kidney, bladder, non-Hodgkin lymphoma, and leukemia. Liver and intrahepatic bile duct cancer is even more common than in the group without DM. On the other hand, although breast cancer is the second most common in the group with DM, its incidence is lower than in the group without DM, as well as prostate cancer.

## Conclusion

In conclusion, this single-institution population-based study shows that CRC patients in complete remission have an increased risk of development of SPMs, especially patients ≥65years of age, with stage I and II primary colon cancer and those with diabetes mellitus. These patients should be frequently and regularly screened for second primary malignancies. This screening should be cheap and without increased radiation load. According to the occurrence of the most common second malignancies, clinical examination, blood count, and ultrasound of the abdomen are appropriate, together with standard breast and colorectal cancer screening, and lung cancer screening under certain conditions, but the frequency of the screening remains unclear.

## Data Availability Statement

The raw data supporting the conclusions of this article will be made available by the authors, without undue reservation.

## Ethics Statement

The studies involving human participants were reviewed and approved by Ethics committee of Masaryk Memorial Cancer Institute. The patients/participants provided their written informed consent to participate in this study.

## Author Contributions

Conceptualization: JH and MS. Data curation: JH and LP. Formal analysis: JH, LP, and TK. Funding acquisition: JH, RD, RG, SK, and TK. Investigation: JH. Methodology: JH, OS, IK, LP, and LB. Project administration: JH. Writing—original draft: JH, TK, and LP. Writing—review and editing: JH, TK, RD, OS, IK, and MS. Supervision, MS. All authors contributed to the article and approved the submitted version.

## Funding

Supported by Ministry of the Health of the Czech Republic, MZ ČR - DRO (MMCI, 00209805) and RI CZECRIN LM2018128 and BBMRI-CZ LM2018125.

## Conflict of Interest

The authors declare that the research was conducted in the absence of any commercial or financial relationships that could be construed as a potential conflict of interest.

## References

[1] Dusek L Pavlík T Májek O Büchler T Muzik J Maluskova D Estimating Cancer Incidence, Prevalence, and the Number of Cancer Patients Treated With Antitumor Therapy in 2015 and 2020 - Analysis of the Czech National Cancer Registry. Klin Onkol (2015) 28(1):30–43. 10.14735/amko201530 25692753

[2] Donin N Filson C Drakaki A Tan HJ Castillo A Kwan L Risk of Second Primary Malignancies Among Cancer Survivors in the United States, 1992 Through 2008. Cancer (2016) 122(19):3075–86. 10.1002/cncr.30164 PMC6192520 27377470

[3] Raj KP Taylor TH Wray C Stamos MJ Zell JA Risk of Second Primary Colorectal Cancer Among Colorectal Cancer Cases: A Population-Based Analysis. J Carcinog (2011) 10:6. 10.4103/1477-3163.78114 21483654 PMC3072650

[4] Vigneri P Frasca F Sciacca L Pandini G Vigneri R Diabetes and Cancer. Endocr Relat Cancer (2009) 16(4):1103–23. 10.1677/ERC-09-0087 19620249

[5] Vigneri R Goldfine ID Frittitta L Insulin, Insulin Receptors, and Cancer. J Endocrinol Invest (2016) 39(12):1365–76. 10.1007/s40618-016-0508-7 27368923

[6] Home P Insulin Therapy and Cancer. Diabetes Care (2013) 36 Suppl 2(Suppl 2):S240–4. 10.2337/dcS13-2002 PMC3920801 23882052

[7] Gallagher EJ LeRoith D Obesity and Diabetes: The Increased Risk of Cancer and Cancer-Related Mortality. Physiol Rev (2015) 95(3):727–48. 10.1152/physrev.00030.2014 PMC4491542 26084689

[8] Thakkar B Aronis KN Vamvini MT Shields K Mantzoros CS Metformin and Sulfonylureas in Relation to Cancer Risk in Type II Diabetes Patients: A Meta-Analysis Using Primary Data of Published Studies. Metabolism (2013) 62(7):922–34. 10.1016/j.metabol.2013.01.014 23419783

[9] Colmers IN Bowker SL Tjosvold LA Johnson JA Insulin Use and Cancer Risk in Patients With Type 2 Diabetes: A Systematic Review and Meta-Analysis of Observational Studies. Diabetes Metab (2012) 38(6):485–506. 10.1016/j.diabet.2012.08.011 23159131

[10] Vajo Z Fawcett J Duckworth WC Recombinant DNA Technology in the Treatment of Diabetes: Insulin Analogs. Endocr Rev (2001) 22(5):706–17. 10.1210/edrv.22.5.0442 11588149

[11] Bordeleau L Yakubovich N Dagenais GR Rosenstock J Probstfield J Chang Yu P The Association of Basal Insulin Glargine and/or n-3 Fatty Acids With Incident Cancers in Patients With Dysglycemia. Diabetes Care (2014) 37(5):1360–6. 10.2337/dc13-1468 24574355

[12] Rosenstock J Fonseca V McGill JB Riddle M Hallé JP Hramiak I Similar Risk of Malignancy With Insulin Glargine and Neutral Protamine Hagedorn (NPH) Insulin in Patients With Type 2 Diabetes: Findings From a 5 Year Randomised, Open-Label Study. Diabetologia (2009) 52(9):1971–3. 10.1007/s00125-009-1452-2 PMC2723677 19609501

[13] Fagot JP Blotière PO Ricordeau P Weill A Alla F Allemand H Does Insulin Glargine Increase the Risk of Cancer Compared With Other Basal Insulins?: A French Nationwide Cohort Study Based on National Administrative Databases. Diabetes Care (2013) 36(2):294–301. 10.2337/dc12-0506 22966091 PMC3554310

[14] Peeters PJ Bazelier MT Leufkens HG Auvinen A van Staa TP de Vries F Insulin Glargine Use and Breast Cancer Risk: Associations With Cumulative Exposure. Acta Oncol (2016) 55(7):851–8. 10.3109/0284186X.2016.1155736 PMC4975082 27150973

[15] Ghosal S Stephens J Van Deventer A Mital V Jayasinghe P Khan M Critical Appraisal of the Recent Data Published on the Link Between Insulin and Cancer. Diabetes Metab Syndr (2011) 5(4):211–3. 10.1016/j.dsx.2012.03.004 25572765

[16] Home PD Lagarenne P Combined Randomised Controlled Trial Experience of Malignancies in Studies Using Insulin Glargine. Diabetologia (2009) 52(12):2499–506. 10.1007/s00125-009-1530-5 PMC2776153 19756478

[17] Wu JW Filion KB Azoulay L Doll MK Suissa S Effect of Long-Acting Insulin Analogs on the Risk of Cancer: A Systematic Review of Observational Studies. Diabetes Care (2016) 39(3):486–94. 10.2337/dc15-1816 26740633

[18] Rendell M Akturk HK Tella SH Glargine Safety, Diabetes and Cancer. Expert Opin Drug Saf (2013) 12(2):247–63. 10.1517/14740338.2013.770469 23394441

[19] Sciacca L Vella V Frittitta L Tumminia A Manzella L Squatrito S Long-acting Insulin Analogs and Cancer. Nutr Metab Cardiovasc Dis (2018) 28(5):436–43. 10.1016/j.numecd.2018.02.010 29609864

[20] Viollet B Guigas B Sanz Garcia N Leclerc J Foretz M Andreelli F Cellular and Molecular Mechanisms of Metformin: An Overview. Clin Sci (Lond) (2012) 122(6):253–70. 10.1042/CS20110386 PMC3398862 22117616

[21] Gonzalez-Angulo AM Meric-Bernstam F Metformin: A Therapeutic Opportunity in Breast Cancer. Clin Cancer Res (2010) 16(6):1695–700. 10.1158/1078-0432.CCR-09-1805 PMC2840206 20215559

[22] Higurashi T Hosono K Takahashi H Komiya Y Umezawa S Sakai E Metformin for Chemoprevention of Metachronous Colorectal Adenoma or Polyps in Post-Polypectomy Patients Without Diabetes: A Multicentre Double-Blind, Placebo-Controlled, Randomised Phase 3 Trial. Lancet Oncol (2016) 17(4):475–83. 10.1016/S1470-2045(15)00565-3 26947328

[23] Kim YH Noh R Cho SY Park SJ Jeon SM Shin HD Inhibitory Effect of Metformin Therapy on the Incidence of Colorectal Advanced Adenomas in Patients With Diabetes. Intest Res (2015) 13(2):145–52. 10.5217/ir.2015.13.2.145 PMC4414756 25931999

[24] Najafi M Cheki M Rezapoor S Geraily G Motevaseli E Carnovale C Metformin: Prevention of Genomic Instability and Cancer: A Review. Mutat Res Genet Toxicol Environ Mutagen (2018) 827:1–8. 10.1016/j.mrgentox.2018.01.007 29502733

[25] Alcusky M Keith SW Karagiannis T Rabinowitz C Louis DZ Maio V Metformin Exposure and Survival in Head and Neck Cancer: A Large Population-Based Cohort Study. J Clin Pharm Ther (2019) 44(4):588–94. 10.1111/jcpt.12820 31293011

[26] Tseng CH Metformin and Lung Cancer Risk in Patients With Type 2 Diabetes Mellitus. Oncotarget (2017) 8(25):41132–42. 10.18632/oncotarget.17066 PMC5522244 28456789

[27] Tseng CH Use of Metformin and Risk of Kidney Cancer in Patients With Type 2 Diabetes. Eur J Cancer (2016) 52:19–25. 10.1016/j.ejca.2015.09.027 26630530

[28] Liu F Yan L Wang Z Lu Y Chu Y Li X Metformin Therapy and Risk of Colorectal Adenomas and Colorectal Cancer in Type 2 Diabetes Mellitus Patients: A Systematic Review and Meta-Analysis. Oncotarget (2017) 8(9):16017–26. 10.18632/oncotarget.13762 PMC5362542 27926481

[29] Coyle C Cafferty FH Vale C Langley RE Metformin as an Adjuvant Treatment for Cancer: A Systematic Review and Meta-Analysis. Ann Oncol (2016) 27(12):2184–95. 10.1093/annonc/mdw410 PMC5178140 27681864

[30] Ding L Liang G Yao Z Zhang J Liu R Chen H Metformin Prevents Cancer Metastasis by Inhibiting M2-like Polarization of Tumor Associated Macrophages. Oncotarget (2015) 6(34):36441–55. 10.18632/oncotarget.5541 PMC4742188 26497364

[31] Decensi A Puntoni M Goodwin P Cazzaniga M Gennari A Bonanni B Metformin and Cancer Risk in Diabetic Patients: A Systematic Review and Meta-Analysis. Cancer Prev Res (Phila) (2010) 3(11):1451–61. 10.1158/1940-6207.CAPR-10-0157 20947488

[32] Vogt A Schmid S Heinimann K Frick H Herrmann C Cerny T Multiple Primary Tumours: Challenges and Approaches, a Review. ESMO Open (2017) 2(2):e000172. 10.1136/esmoopen-2017-000172. eCollection 2017. 28761745 PMC5519797

[33] SEER Training Modules Multiple primary neoplasms. U. S. National Institutes of Health: National Cancer Institute Available at: https://training.seer.cancer.gov/. [Retrieved June 10, 2020]

[34] Working Group Report International Rules for Multiple Primary Cancers (ICD-0 Third Edition). Eur J Cancer Prev (2005) 14(4):307–8. 10.1097/00008469-200508000-00002 16030420

[35] Institute of Health Information and Statistics of the Czech Republic National Health Information System (NHIS), Czech National Cancer Registry (CNCR). http://www.uzis.cz/en/czech-nationalcancer-registry-cncr. [Retrieved June 10, 2020]

[36] Jia H Li Q Yuan J Sun X Wu Z Second Primary Malignancies in Patients With Colorectal Cancer: A Population-Based Analysis. Oncologist (2020) 25 (4):e644–50. 10.1634/theoncologist.2019-0266 PMC7160402 31943509

[37] La Francis IE Cooper RB Second Primary Malignancies Associated With Primary Female Breast Cancer: A Review of the Danbury Hospital Experience. Conn Med (1992) 56(8):411–4. 1526142

[38] Duchateau CS Stokkel MP Second Primary Tumors Involving Non-Small Cell Lung Cancer: Prevalence and Its Influence on Survival. Chest (2005) 127 (4):1152–8. 10.1378/chest.127.4.1152 15821189

[39] Lee YT Liu CJ Hu YW Teng CJ Tzeng CH Yeh CM Incidence of Second Primary Malignancies Following Colorectal Cancer: A Distinct Pattern of Occurrence Between Colon and Rectal Cancers and Association of Co-Morbidity With Second Primary Malignancies in a Population-Based Cohort of 98,876 Patients in Taiwan. Medicine (Baltimore) (2015) 94(26):e1079. 10.1097/MD.0000000000001079 26131831 PMC4504576

[40] Takeuchi D Koide N Komatsu D Okumura M Suzuki A Miyagawa S Relationships of Obesity and Diabetes Mellitus to Other Primary Cancers in Surgically Treated Gastric Cancer Patients. Int J Surg (2014) 12(6):587–93. 10.1016/j.ijsu.2014.04.012 24802517

[41] Broman KK Bailey CE Parikh AA Sidedness of Colorectal Cancer Impacts Risk of Second Primary Gastrointestinal Malignancy. Ann Surg Oncol (2019) 26(7):2037–43. 10.1245/s10434-019-07326-7 30949861

[42] Li CI Daling JR Tang MT Malone KE Relationship between diabetes and risk of second primary contralateral breast cancer. Breast Cancer Res Treat (2011) 125(2):545–51. 10.1007/s10549-010-1035-4 PMC3005863 20625814

[43] Zhuang L Yan X Meng Z Second primary malignancy in patients with cholangiocarcinoma: a population-based study. Cancer Manag Res (2019) 11:1969–83. 10.2147/CMAR.S187614 PMC6402443 30881122

[44] González N Prieto I Del Puerto-Nevado L Portal-Nuñez S Ardura JA Corton M 2017 Update on the Relationship Between Diabetes and Colorectal Cancer: Epidemiology, Potential Molecular Mechanisms and Therapeutic Implications. Oncotarget (2017) 8(11):18456–85. 10.18632/oncotarget.14472 PMC5392343 28060743

[45] Morden NE Liu SK Smith J Mackenzie TA Skinner J Korc M Further Exploration of the Relationship Between Insulin Glargine and Incident Cancer: A Retrospective Cohort Study of Older Medicare Patients. Diabetes Care (2011) 34(9):1965–71. 10.2337/dc11-0699 PMC3161263 21775752

[46] Kwon M Roh JL Song J Lee SW Kim SB Choi SH Effect of Metformin on Progression of Head and Neck Cancers, Occurrence of Second Primary Cancers, and Cause-Specific Survival. Oncologist (2015) 20(5):546–53. 10.1634/theoncologist.2014-0426 PMC4425387 25802404

[47] Zhou PT Li B Liu FR Zhang MC Wang Q Li YY Metformin Is Associated With Survival Benefit in Pancreatic Cancer Patients With Diabetes: A Systematic Review and Meta-Analysis. Oncotarget (2017) 8(15):25242–50. 10.18632/oncotarget.15692 PMC5421925 28445955

[48] Bradley MC Ferrara A Achacoso N Ehrlich SF Quesenberry CP Jr Habel LA A Cohort Study of Metformin and Colorectal Cancer Risk Among Patients With Diabetes Mellitus. Cancer Epidemiol Biomarkers Prev (2018) 27(5):525–30. 10.1158/1055-9965.EPI-17-0424 PMC5935125 29716927

[49] Dankner R Balicer R Boffetta P Boker LK Wallenstein S Freedman L Diabetes, Glucose Control, Glucose Lowering Medications, and Cancer Risk: A 10-year Population-Based Historical Cohort. BMC Cancer (2012) 12:364. 10.1186/1471-2407-12-364 22917080 PMC3488338

[50] Ruiter R Visser LE van Herk-Sukel MP Coebergh JW Haak HR Geelhoed-Duijvestijn PH Risk of cancer in patients on insulin glargine and other insulin analogs in comparison with those on human insulin: results from a large population-based follow-up study. Diabetologia (2012) 55:51–62. 10.1007/s00125-011-2312-4 21956710 PMC3228952

[51] Karlstad O Starup-Linde J Vestergaard P Hjellvik V Bazelier MT Schmidt MK Use of Insulin and Insulin Analogs and Risk of Cancer - Systematic Review and Meta-Analysis of Observational Studies. Curr Drug Saf (2013) 8(5):333–48. 10.2174/15680266113136660067 PMC3899599 24215311

[52] Mayer D Chantelau E Treatment With Insulin Glargine (Lantus) Increases the Proliferative Potency of the Serum of Patients With type-1 Diabetes: A Pilot Study on MCF-7 Breast Cancer Cells. Arch Physiol Biochem (2010) 116(2):73–8. 10.3109/13813451003631439 20199195

[53] But A De Bruin ML Bazelier MT Hjellvik V Andersen M Auvinen A Cancer Risk Among Insulin Users: Comparing Analogues With Human Insulin in the CARING Five-Country Cohort Study. Diabetologia (2017) 60(9):1691–703. 10.1007/s00125-017-4312-5 PMC5552833 28573394

[54] Gibson TM Park Y Robien K Shiels MS Black A Sampson JN Body Mass Index and Risk of Second Obesity-Associated Cancers After Colorectal Cancer: A Pooled Analysis of Prospective Cohort Studies. J Clin Oncol (2014) 32(35):4004–11. 10.1200/JCO.2014.56.8444 PMC4251963 25267739

[55] Morais S Castro C Antunes L Peleteiro B Bento MJ Lunet N Second Primary Cancers and Survival in Patients With Gastric Cancer: Association With Prediagnosis Lifestyles. Eur J Cancer Prev (2019) 28(3):159–66. 10.1097/CEJ.0000000000000447 29668653

[56] Wood ME Vogel V Ng A Foxhall L Goodwin P Travis LB Second Malignant Neoplasms: Assessment and Strategies for Risk Reduction. J Clin Oncol (2012) 30(30):3734–45. 10.1200/JCO.2012.41.8681 23008293

